# The metabolic and autoregulatory profile of reversible delayed cerebral ischemia in unconscious patients after aneurysmal subarachnoid hemorrhage: a prospective multimodal neuromonitoring cohort study

**DOI:** 10.1186/s13054-025-05460-1

**Published:** 2025-06-05

**Authors:** Michael Veldeman, Stefan Yu Bögli, Ihsane Olakorede, Nick Kastenholz, Miriam Weiss, Catharina Conzen-Dilger, Katharina Seyfried, Erta Beqiri, Charlotte Weyland, Hans Clusmann, Gerrit Alexander Schubert, Anke Hoellig, Peter Smielewski

**Affiliations:** 1 Department of Neurosurgery, RWTH Aachen University Hospital, Pauwelsstrasse 30, 52074 Aachen, Germany; 2 Brain Physics Laboratory, Division of Neurosurgery, Department of Clinical Neurosciences, University of Cambridge, Cambridge, UK; 3 Department of Neurology and Neurocritical Care Unit, Clinical Neuroscience Center, University Hospital and University of Zurich, Zurich, Switzerland; 4 Department of Neurosurgery, Faculty of Medicine and University Hospital, Center for Neurosurgery, University of Cologne, Cologne, Germany; 5 Department of Neurosurgery, Cantonal Hospital Aarau, Aarau, Switzerland; 6 Faculty of Medicine, University of Bern, Bern, Switzerland; 7 Department of Diagnostic and Interventional Neuroradiology, RWTH Aachen University Hospital, Aachen, Germany

**Keywords:** Subarachnoid hemorrhage, Delayed cerebral ischemia, Cerebral microdialysis, Cerebral autoregulation, Perfusion computed tomography, Cerebral infarction

## Abstract

**Background:**

The detection and treatment of delayed cerebral ischemia (DCI) following aneurysmal subarachnoid hemorrhage (SAH) remain challenging. Multimodal neuromonitoring and CT perfusion scanning (CTP) are promising tools for diagnosing DCI in unconscious patients. This study aims to compare the metabolic and autoregulatory characteristics of patients with cerebral hypoperfusion indicative of DCI that either resolves post-treatment or progresses to infarction due to treatment failure.

**Methods:**

In a cohort of 268 consecutive SAH patients, neuromonitoring—comprising intracranial pressure (ICP) and brain tissue oxygen (PtiO_2_) measurements, and/or cerebral microdialysis—was implemented in 126 (47%) neurologically unassessable patients. Aberrant neuromonitoring measurements triggered CTP, and in cases of confirmed perfusion deficits, first-tier treatment involved induced hypertension. Non-responsive perfusion deficits were further evaluated with conventional angiography, and spasmolysis or angioplasty was performed if suitable vasospasm was identified. DCI-related infarction was noted on CT imaging at discharge, and clinical outcomes were assessed using the modified rankin scale (mRS) at 12 months. Using a generalized linear mixed-effects model (GLMM), factors associated with the occurrence of DCI-related infarction were assessed.

**Results:**

CTP deficits were identified in 72 (57%) patients, of whom 63 (88%) had neuromonitoring probes near the affected areas. In 24 patients (38%), perfusion deficits progressed to infarction, while in 39 (62%), deficits were successfully reversed through induced hypertension or spasmolysis. In a GLMM, lower pressure reactivity index (PRx–OR 2.70, 95% CI 1.04–4.67; *p* < 0.001) and lower lactate-to-pyruvate ratio (LPR–OR 1.02, 95% CI 1.01–1.03; *p* < 0.001) were independently associated with better treatment response and reduced infarction risk, after adjusting for clinical hemorrhage severity. These effects were observed more than 24 h before cerebral hypoperfusion. Pooled PRx and LPR over this time frame were not associated with functional outcome.

**Conclusion:**

Loss of cerebrovascular reactivity and metabolic disturbances precede cerebral hypoperfusion in SAH. Lower PRx and LPR levels are independently associated with improved DCI treatment efficacy. These findings must be interpreted in the context of study limitations, including the small sample size and the focal nature of microdialysis measurements. Nevertheless, the results suggest that invasive neuromonitoring may aid in identifying patients more likely to benefit from treatment.

**Trial registration:**

This project was retrospectivly registered in the German Clinical Trial Register (DRKS00030505) on the third of January 2023.

**Supplementary Information:**

The online version contains supplementary material available at 10.1186/s13054-025-05460-1.

## Introduction

Aneurysmal subarachnoid hemorrhage (SAH) affects approximately 9 individuals per 100,000 annually and poses a significant socio-economic burden due to its occurrence in relatively young patients [1, 2]. Beyond initial clinical and radiological severity, outcome is determined by the development of delayed cerebral ischemia (DCI) [3]. DCI is a multifactorial, potentially reversible mismatch between oxygen and glucose delivery and metabolic demand. Supply is reduced by angiographic and microvascular vasospasm, microthrombosis, inverted neurovascular coupling and impaired autoregulation, while demand increases due to neuroinflammation and cortical spreading depolarizations [4, 5]. Without effective treatment, DCI progresses to cerebral infarction, compromising functional outcome [3, 6]. 

Timely diagnosis of DCI remains challenging. Whilst broadly adapted definitions for DCI exist in neurologically assessable patients [7], standardization in unconscious patients is lacking. Approximately 20–30% of SAH patients present in an unconscious state upon hospital admission [8]. Furthermore, about 20% of patients experience secondary neurological deterioration during the period of highest vulnerability to DCI [9].

Multimodal neuromonitoring offers potential for early detection of DCI-related pathophysiological changes, allowing further diagnostic exploration [10, 11]. Detection tools for DCI are typically evaluated by their temporal and spatial resolution. Invasive techniques such as brain tissue oxygen (PtiO_2_) monitoring and cerebral microdialysis (µD) provide excellent temporal resolution with real-time bedside data. However, these methods are limited to the local tissue surrounding the probe tip [12]. In contrast, perfusion CT (CTP) imaging, increasingly used for diagnosing hemodynamically relevant vasospasm, offers spatial coverage of all cerebrovascular territories. Despite its low temporal resolution, CTP provides critical insights into perfusion deficits that are often interpreted and treated as DCI [13].

Autoregulation, frequently impaired in SAH [14, 15], can be assessed using the pressure reactivity index (PRx). Higher PRx values, indicative of autoregulatory dysfunction, are associated with poorer functional outcomes after SAH [16, 17]. Parameters derived from neuromonitoring during the early phase (first 72 h) have been linked to clinical outcome [18, 19]. Metabolic markers, such as glucose, lactate, and glutamate, reflect the severity of SAH and excitotoxicity [20]. Among these, the lactate-to-pyruvate ratio (LPR) has shown the strongest association with DCI and poor 12-month functional outcomes [21, 22]. High LPR and low glucose precede CT hypoperfusion [23] but to what extent monitoring results might predict treatment effect, has not been investigated yet.

### Objectives

This study aims to compare metabolic and autoregulatory characteristics of patients with hypoperfusion indicative of DCI that either resolves post-treatment or progresses to infarction due to treatment failure. The goal is to develop a predictive model based on neuromonitoring parameters to identify patients most likely to benefit from DCI treatment. Herein, we hypothesize that SAH patients with intact autoregulation, exhibiting a rightward shift in the autoregulatory curve, respond more effectively to mild-to-moderate induced hypertension or macrovasodilatory treatment. Conversely, in patients with impaired autoregulation, these interventions may be unsuccessful or even harmful. Additionally, we hypothize that severe metabolic disturbances in hypoperfused brain regions may indicate irreversibly advanced DCI.

## Methods

This is an observational study, conducted on a prospective cohort of SAH patients. Strengthening the Reporting of Observational Studies in Epidemiology (STROBE) guidelines were followed in the writing of this text [24].

### Standard protocol approval, registration, and patient consent

The local ethics committee of the medical faculty at RWTH Aachen University approved the data collection (EK 14/062 and EK 22/371). Informed consent was obtained from all included patients.

### Participants

A prospective registry of all consecutive SAH patients treated at RWTH Aachen University Hospital, Germany, has been maintained since 2014. Aneurysm rupture was confirmed through either CT- or conventional angiography. Patients aged 18 years or older treated until 2020, who underwent neuromonitoring, were considered for inclusion. Clinical hemorrhage severity was classified based on the best glasgow coma scale (GCS) score [25] within the first 24 h and graded according to the world federation of neurological surgeons (WFNS) grading scale [26]. Clinical severity was dichotomized into good-grade (WFNS 1–2) and poor-grade (WFNS 3–5) SAH. Radiological severity was assessed using the modified Fisher scale (mFisher) [27].

### Standard treatment algorithm

Patients were managed according to a standardized protocol published previously [28, 29]. Aneurysms were secured early, within 24 h, via clip ligation or endovascular embolization, followed by monitoring in a specialized neurointensive care unit. Acute hydrocephalus was treated with external ventricular drainage (EVD), and all patients received prophylactic oral nimodipine.

This study focuses on patients who were either neurologically not assessable upon admission or experienced secondary deterioration due to DCI or other causes (e.g. need for sedation), rendering them neurologically unexaminable. These patients received invasive neuromonitoring unless contraindicated by coagulation disorders or predictable early mortality. PtiO_2_ probes (Neurovent PTO®, Raumedic, Germany) and µD catheters (71 High Cut-Off Brain Microdialysis Catheter, µdialysis, Sweden) were positioned on the side of the ruptured aneurysm or, in midline aneurysms, on the side with greater subarachnoid blood load. Probes were positioned via a double-bold in the frontal watershed region, 4.5 cm lateral to the midline, anterior to the coronal suture, to monitor both middle and anterior cerebral artery territories.

DCI diagnosis relied on neuromonitoring, where transcranial Doppler (TCD) vasospasm (mean flow velocity > 120 cm/s), cerebral oxygenation crises (PtiO_2_ < 20 mmHg), or metabolic derangements (LPR > 40) triggered CTP. Radiologically confirmed DCI was defined as territorial or watershed perfusion deficits on CTP with a time to drain > 10 s and mean transit time > 6.7 s [30].

After radiological confirmation, treatment was initiated. First tier treatment consisted of inducing euvolemic hypertension through norepinephrine infusion. Treatment was typically reevaluated with CTP, 6–12 h after starting treatment. If perfusion mismatch persisted, second-tier endovascular rescue therapies were discussed and implemented after reaching interdisciplinary consensus. Angioplasty was utilized for proximal vasospasm, while intra-arterial spasmolysis was applied in cases of diffuse vasospasm. Our institutional endovascular DCI treatment protocol has been published previously and allows for continuous and / or bilateral spasmolysis by intra-arterial nimodipine infusion [31]. Regardless of the treatment tier, therapy was reviewed daily and scaled back as soon as clinically appropriate.

### Study design

This study consists of a cohort analysis comparing neuromonitoring parameters in two groups of patients with either CTP deficits who were successfully treated (treatment responders) *versus* those in whom treatment failed, resulting in DCI-related infarction of the hypoperfused area (non-responders). The study aims to assess potential associations between pre-DCI cerebral autoregulation and metabolism, in relation to treatment response.

**Fig. 1:**
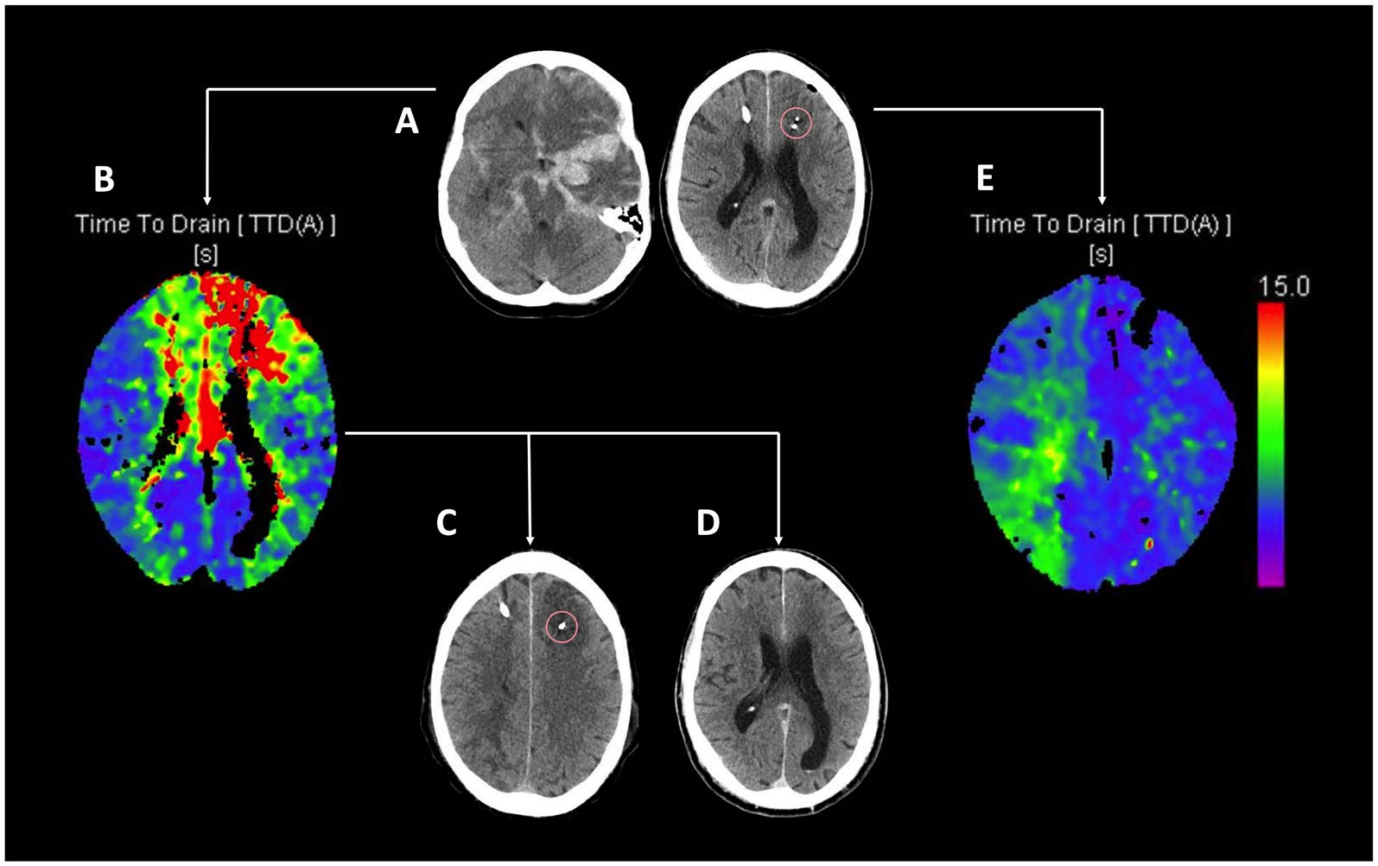
Exemplary depiction of the spatial relation between perfusion deficits and invasive neuromonitoring probes as a prerequisite for study inclusion. **A** Aneurysmal subarachnoid hemorrhage with left-sided blood distribution provided with left-sided dual invasive neuromonitoring (PtiO_2_ and cerebral microdialysis) in the frame on the right. **B** Bifrontal, predominantly left-sided perfusion deficits developed in an area covered by the probes. Patients with this constellation, were included in the study. **C** The affected area evolves into cerebral infarction despite treatment. Patients with this course of events were included in the study group with infarction. **D** The affected area is spared from infarction due to successful treatment. These patients were included as the no infarction group. **E** Patients with perfusion deficits outside the reach of invasive neuromonitoring were excluded from this analysis. PtiO_2_, brain tissue oxygen pressure

CTP examinations of all patients were evaluated, and the time point of the first identified perfusion deficit (in hours from reconstructed aneurysm rupture) was recorded. In subsequent imaging following treatment initiation, possible demarcation of the hypoperfused area was noted and classified as DCI-related infarction [6]. Infarction was diagnosed independently by two assessors, and any discrepancies were resolved through discussion until consensus was reached. The spatial relationship between invasive neuromonitoring probes and the region of hypoperfusion was evaluated. A probe was considered to be within or adjacent to the affected region of interest if the hypoperfused area was located in the ipsilateral A1, A2, M1, M4, or M5 regions, as defined by the extended Alberta Stroke Program Early CT Score (ASPECTS) [32] as described previously [6]. This methodology is visually depicted in Fig. 1 and is more liberal compared to previous similar analyses [28].

All longitudinal data was timely anchored to the hour of initial CTP hypoperfusion, aligning 60 min binned physiological data (CPP, PRx and PtiO_2_) with µD values. Further analyses focused on neuromonitoring results preceding perfusion deficits.

### Monitoring and data acquisition

Arterial blood pressure was measured invasively using a standard pressure transducer. Intracranial pressure (ICP) and brain tissue oxygen tension (PtiO_2_) were registered using the Neurovent-PTO® intraparenchymal probe (Raumedic, Helmbrechts, Germany). High-frequency waveform data were collected for mean arterial blood pressure (MAP) and ICP at 100 Hz, while PtiO_2_ was recorded at 1 Hz. Data were stored using the MPR2 logO Datalogger (Raumedic, Helmbrechts, Germany) until July 2018, after which the Moberg CNS monitor (Component Neuromonitoring System, Moberg Research, Ambler, PA) was utilized. Data from the initial calibration phase, following the insertion of probes, were manually excluded.

Clinical outcomes were assessed 12 months post-hemorrhage using the modified Rankin Scale (mRS), either through in-person evaluations or via structured telephone interviews of patients or their next of kin [33]. The mRS was dichotomized into favorable (mRS 0–3, independent) and unfavorable (mRS 4–6, dependent) outcome.

Microdialysis catheters were perfused at a rate of 0.3 μl/min using a standard crystalloid solution (CNS perfusion fluid, µdialysis, Sweden). Collection vials were replaced every three hours. The frequency increased to hourly intervals if abnormal results were detected. The dialysate was analyzed bedside using ISCUSflex® (µdialysis, Sweden) to measure levels of lactate (mmol/L), pyruvate (μmol/L), glucose (mmol/L), glutamate (μmol/L), and glycerol (mmol/L).

### Data processing

Artifacts were removed manually and indices computed using ICM + multimodal monitoring software (University of Cambridge, Cambridge Enterprise, Cambridge, United Kingdom). The pressure reactivity index (PRx) was used to assess cerebrovascular reactivity [34]. ICP and MAP values were averaged over 10-s intervals. PRx was determined as the Pearson correlation coefficient calculated over a 5-min moving window (with 80% overlap) [35]. Physiological variables were averaged over 60-min intervals for the final analysis.

### Statistical analysis

All statistical analyses were conducted and graphics were created using R (v. 4.4.0; www.r-project.org) in RStudio (v. 2024.12.0 + 467). Analyses were performed using packages from the *tidyverse*, along with *broom*, *car*, *DHARMa*, *lme4*, *mice*, mgcv, *performance*, *smooth*, and *vioplot*. Quantitative variables were assessed for normality through histograms and quantile–quantile plots and, where necessary, the Shapiro–Wilk test. Normally distributed numeric variables are reported as mean ± standard deviation (SD) and were compared between groups using unpaired T-tests. Non-parametric data are reported as medians with interquartile ranges (Q1–Q3) and analyzed using Mann–Whitney U tests. Categorical variables are expressed as proportions (%) and tested using χ^2^ tests or Fisher’s exact tests in cases of sparse data.

Exploratory analyses to identify the time frame of interest preceding CTP hypoperfusion were conducted by plotting the mean and standard deviation (SD), or median and interquartile range (IQR; Q1 to Q3), of CPP, PRx, PtiO_2_, and µD data, as appropriate, over time per hour up to the first CTP hypoperfusion event. Additionally, a Locally Estimated Scatterplot Smoothing (Loess) curve with 95% confidence intervals (CIs) was plotted to visualize trends over a 7-day (168 h) time window.

For the selection of predictors for modeling DCI treatment response, three methods were employed. Firstly, pooled variables over the selected time frame were compared using univariate statistical analyses. To correct for pseudoreplication in this time series data, a single mean or median (as appropriate) value per patient was used. Secondly, given the non-linear relationships observed, Generalized Additive Mixed Models (GAMMs) were implemented to model neuromonitoring data over time. The methodology was adapted from previous similar work in traumatic brain injury [36]. Patient-level random effects were introduced, and smooth terms were constructed using thin-plate regression splines, accompanied by a continuous-time autoregressive term. The smooth terms were penalized using the Restricted Maximum Likelihood (REML) method. Nested models of hourly temporal trends, with or without the addition of infarct development as an input variable, were compared using a Likelihood Ratio Test (LRT) for each individual potential predictor. Thirdly, for further analysis of hourly data, a Generalized Linear Mixed-Effects Model (GLMM) was developed using a forward selection approach. Modeling began with a null model that included only a random intercept at the individual level to control for repeated measurements or clustering within individuals. Predictor variables, deemed informative based on plotting and univariate results, were then added to the model in a stepwise manner. Variables were tested for multicollinearity via assessment of the Variance Inflation Factor with a cut-off of 2.5. At each step, model performance was evaluated using the Akaike Information Criterion (AIC), where a decrease indicated an improved model fit. Additionally, LRTs were applied to compare nested models of increasing complexity. The final model included only predictors that demonstrated a significant contribution to the model fit based on these criteria or where necessary to correct for pseudoreplication.

To address issues of stability and convergence in the GLMM, a combination of data imputation and scaling strategies was applied. Missing data were handled using multiple imputations via a Fully Conditional Chained Equation model, generating 5 imputed datasets. The missing data rate was evaluated for each variable, and imputation was performed only if less than 5% of observations were missing. Each dataset was entered into a GLMM, and results were pooled to obtain overall estimates of odds ratios (ORs) and confidence intervals (CIs) [37]. Furthermore, prior to inclusion, µD concentrations were scaled to a standard normal distribution to improve numerical stability. After model estimation, ORs and CIs were rescaled to their original units.

Dichotomized clinical outcome was assessed in a logistic regression model including predictors via forward selection in analogy with the GLMM. Statistical significance was defined as a two-sided *p*-value < 0.05.

## Results

### Patients’ inclusion and demographics

**Fig. 2:**
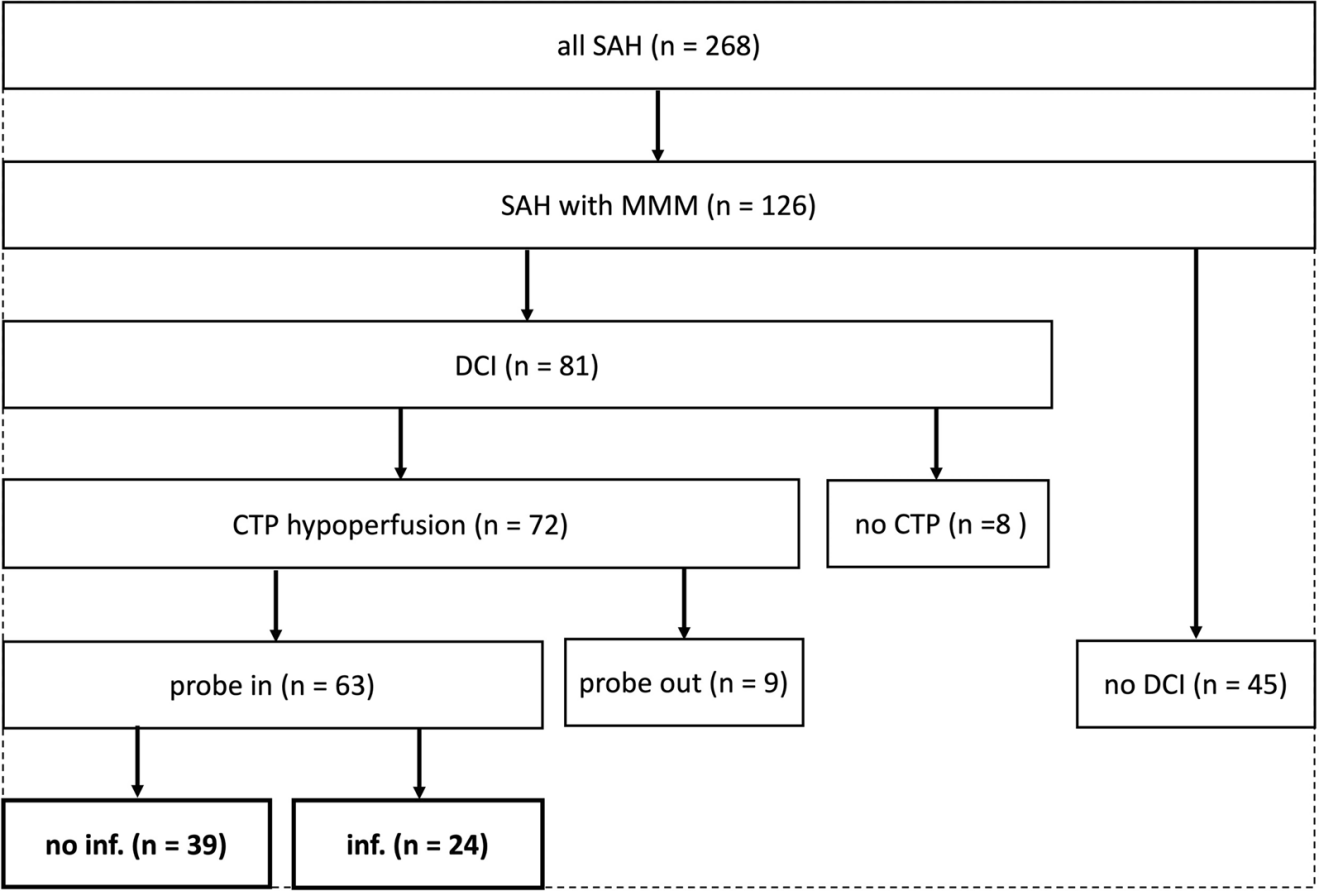
Inclusion flow chart. CTP, perfusion computed tomography imaging; DCI, delayed cerebral ischemia; inf., cerebral infarction; MMM, multimodal monitoring; SAH, aneurysmal subarachnoid hemorrhage

During the inclusion period, a total of 268 SAH patients were admitted, of whom 126 (47.0%) underwent ICP and PtiO_2_ monitoring, of which an additional 97 (36.2%) received cerebral microdialysis. Monitoring was placed a median of one day (IQR; 0 to 3) after SAH. We compared the absolute and relative (%) numbers of patients receiving invasive neuromonitoring per modality throughout the study period (2014–2020) to assess potential selection bias (Additional file 1: Table S1). While variability in annual monitoring rates was observed, this likely reflects epidemiological variation in patient presentation rather than a lowered threshold for monitoring application. Among patients with neuromonitoring, 81 patients received treatment for DCI, with the decision to treat based on a CTP perfusion deficit in 72 (57.1%) cases. Invasive neuromonitoring probes were located within or adjacent to the hypoperfused area in 63 (87.5%) of those patients, thus these were included in the analysis. Finally, out of those, hypoperfused regions, 24 (38.1%) progressed to cerebral infarction. A flowchart illustrating patient inclusion is provided in Fig. 2.

The mean age of the 63 patients included in the final analysis was 54.7 ± 11.8 years, with 71.4% being female. Age and sex distributions were comparable between patients with a positive treatment response and those who developed infarction. Similarly, neither the clinical nor radiological hemorrhage severity was associated with treatment response. A detailed overview of patient and hemorrhage-specific characteristics is presented in Table 1.

**Table 1:** Baseline and hemorrhage specific characteristics of included subarachnoid hemorrhage patients in relation to whether detected perfusion deficits evolved into DCI-related infarction

	All	No DCI infarction	DCI infarction	Univariate *p*-value
	n = 63	n = 39	n = 24	
*Demographics*				
Age—yrs.—mean ± SD (range)	54.7 ± 11.8 (19–87)	56.0 ± 11.6 (32–87)	52.6 ± 12.0 (19–73)	0.267
Sex—female / male—no. (%)	45 (71.4)/18 (28.6)	26 (66.7)/13 (33.3)	19 (79.2)/5 (20.8)	0.426
*Monitoring*				
Overall duration (days)	17.0 ± 7.8	16.8 ± 7.6	17.5 ± 8.2	0.745
Lag to CTP deficit (hrs)	176.0 ± 74.2	165.0 ± 71.3	196 ± 76.7	0.107
*Aneurysm location—no. (%)*				
Acomm	27 (42.9)	16 (41.0)	11 (45.8)	0.913
MCA	11 (17.5)	6 (15.4)	5 (20.8)	
ICA (inc. Pcomm)	14 (22.2)	9 (23.1)	5 (20.8)	
BA	3 (4.8)	2 (5.1)	1 (4.2)	
Others	8 (12.7)	6 (15.4)	2 (8.3)	
Ant. circulation	56 (88.9)	35 (89.7)	21 (87.5)	0.711
*Aneurysm occlusion—no. (%)*				
Clipping / endovascular	24 (38.0)/39 (62.0)	13 (47.4)/26 (52.6)	11 (45.8)/13 (54.2)	0.598
*Hemorrhage severity*				
WFNS grade—no. (%)				0.622
Grade 1	11 (17.5)	7 (17.9)	4 (16.7)	
Grade 2	6 (9.5)	5 (12.8)	1 (4.2)	
Grade 3	15 (23.8)	10 (25.6)	5 (20.8)	
Grade 4	17 (27.0)	8 (20.5)	9 (37.5)	
Grade 5	14 (22.2)	9 (23.1)	5 (20.8)	
Poor-grade SAH (WFNS 3–5)	46 (73.0)	27 (69.2)	19 (79.2)	0.560
Modified Fisher scale—no. (%)				0.640
Grade 1	8 (12.7)	5 (12.8)	3 (12.5)	
Grade 2	8 (12.7)	6 (15.4)	2 (8.3)	
Grade 3	16 (25.4)	8 (20.5)	8 (33.3)	
Grade 4	31 (49.2)	20 (51.3)	11 (45.8)	
Intracerebral hemorrhage	27 (42.9)	18 (46.2)	9 (37.5)	0.680
*DCI treatment*				
Induced hypertension—no. (%)	58 (92.1)	36 (92.3)	22 (91.7)	0.990
Spasmolysis—no. (%)	36 (57.1)	22 (56.4)	14 (58.3)	0.780
Angioplasty—no. (%)	9 (14.3)	4 (10.3)	5(20.8)	0.257
*Clinical outcome*				
mRS 12 months—no. (%)				0.282
No symptoms	5 (7.9)	3 (7.7)	2 (8.3)	
No significant disability	5 (7.9)	5 (12.8)	0	
Slight disability	6 (9.5)	4 (10.3)	2 (8.3)	
Moderate disability	6 (9.5)	3 (7.7)	3 (12.5)	
Moderate severe disability	9 (14.3)	7 (17.9)	2 (8.3)	
Severe disability	6 (9.5)	4 (10.3)	2 (8.3)	
Dead	19 (30.2)	8 (20.8)	11 (45.8)	
Favorable outcome (mRS 0–3)	22 (34.9)	15 (38.5)	7 (29.2)	0.398
Missing	7 (11.1)	5 (12.8)	2 (8.3)	0.699

Acomm, anterior communicating artery; ant., anterior; BA, basilar artery; GOS, Glasgow outcome scale; ICA, internal carotid artery; inc., including; MCA, middle cerebral artery; mFisher, modified Fisher scale; mRS, modified Rankin scale; Pcomm, posterior communicating artery; Q1, first quartile; Q3, third quartile; SAH, aneurysmal subarachnoid hemorrhage; SD, standard deviations; WFNS, World Federation of Neurosurgical Societies grading; yrs., years

### Identification of the time window of interest

Scatterplots of 7 days of data, with Loess line fitting demonstrated a divergence in lactate-to-pyruvate ratio (LPR) curves approximately 50 h before CTP hypoperfusion, as well as for glutamate levels around 60 h prior (Additional file 1: Fig. S1). Higher LPR and glutamate levels were observed in patients where treatment was unsuccessful. PRx curves showed an increase in both treatment responders and infarct developers starting around 50 h before hypoperfusion, with higher values in the latter group. Additionally, a decrease in PtiO_2_ was observed in both groups up to 100 h prior to hypoperfusion; however, levels stabilized thereafter in both groups. The corresponding plots are shown in Additional file 1: Fig. S1.

A time window of 72 h prior to CTP hypoperfusion was deemed most appropriate with LPR, PRx and glutamate demonstrating divergent higher levels in non-responders to treatment within this time frame (Additional file 1: Fig. S2).

### Modelling temporal trends

**Fig. 3:**
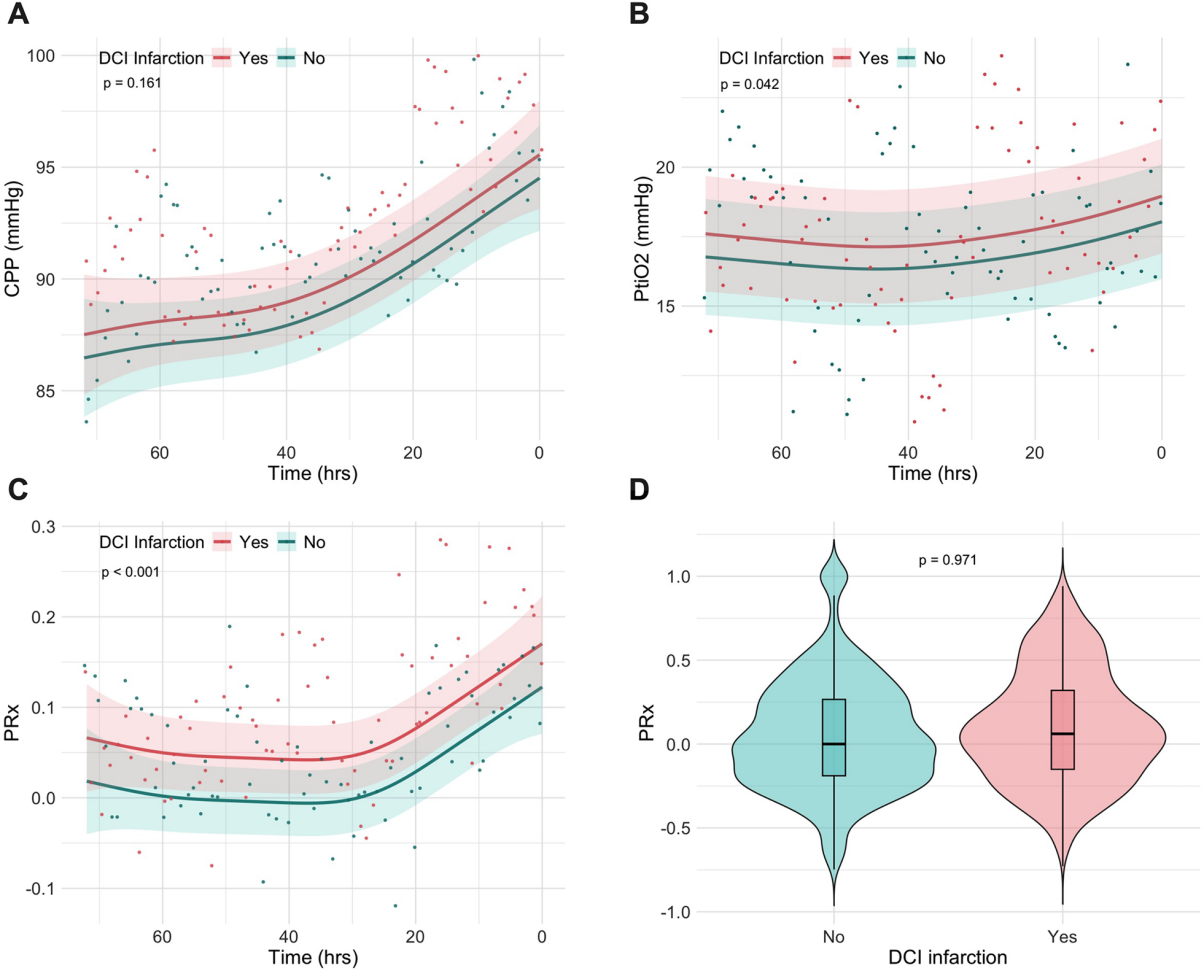
Generalized additive mixed model (GAMM) predicted time courses for **A** cerebral perfusion pressure (CPP), **B** brain tissue oxygen pressure (PtiO_2_ (mmHg)) pressure and **C** pressure reactivity index (PRx); 72 h before radiological confirmation of DCI and treatment initiation. Predictions have been split by infarction status. Shaded areas indicated 95% confidence intervals. The superimposed scatterplot represents raw data expressed as hourly mean (CPP & PRx) or hourly median (PtiO_2_) values. **D** Individually averaged PRx over 72 h of time split by infarction status. Reported p-values in panel A, B & C represent Likelihood Ratio Test results for nested models with or without DCI infarction as a predictor. In panel D, the *p*-value represents the result of an independent T-test performed on patient values averaged over the 72-h time window. DCI, delayed cerebral ischemia; hrs, hours; mmHg, millimeters of mercury

**Fig. 4:**
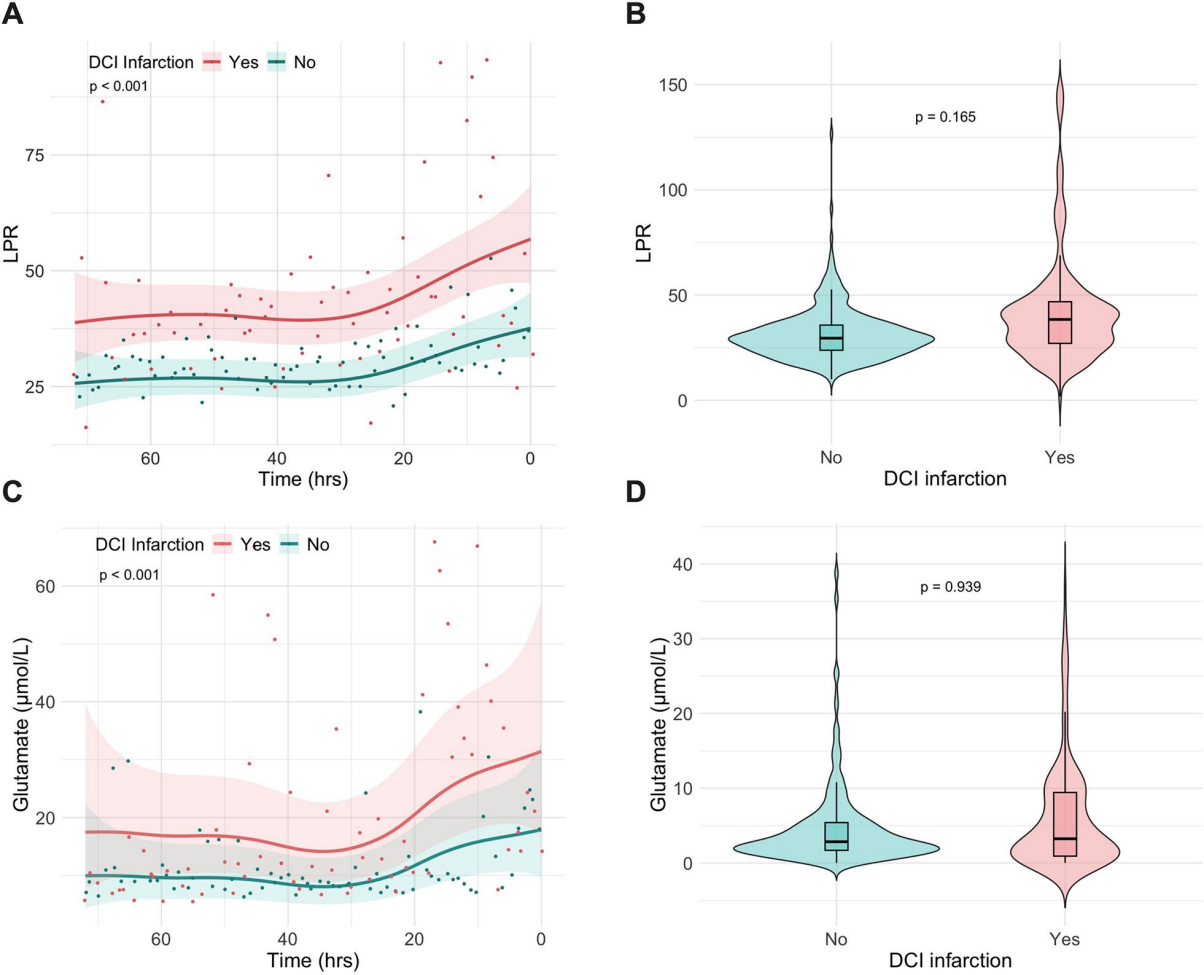
Generalized Additive Mixed Model (GAMM) predicted time courses of microdialysis analytes **A** lactate-to-pyruvate ratio (LPR), **B** individual median LPR over 72 h of time split by infarction status, **C** glutamate (µmol/L), **D** individual median glutamate concentrations over 72 h of time split by infarction status. Shaded areas indicated 95% confidence intervals. The superimposed scatterplot represents raw data expressed as one median per hour. Reported *p*-values in panel A & C represent Likelihood Ratio Test results for nested models with or without DCI infarction as a predictor. In panel B & C, the *p*-value represents the result of a Mann–Whitney U tests performed on a data consisting of a single median per patient over the 72-h time window. DCI, delayed cerebral ischemia; hrs, hours; LPR, lactate-to-pyruvate ratio

GAMMs were constructed to fit the temporal profile of CPP, PRx, PtiO_2_ and microdialysis analytes, 72 h preceding CTP hypoperfusion. Curves were stratified by treatment response (see Figs. 3 and 4). Predicted values from the model were plotted over time with 95% CIs, to visualize the temporal trends and the effect of the stratifying variable. CPP increased similarly between outcome groups over time, potentially reflecting spontaneous permissive hypertension [38]. PRx increased over time in both groups and was generally higher in the DCI infarction group. Infarction status significantly improved model fit (*p* < 0.001). However, pooled PRx means between treatment response groups—while accounting for repeated measurements but not time trends—did not differ significantly (*p* = 0.971).

GAMM predictions of PtiO₂ largely overlapped between the treatment response groups. GAMM predictions of CPP, PtiO_2_ and PRx over time, are presented in Fig. 3. A dissociation between LPR and glutamate concentrations over time was observed, with non-responders exhibiting higher LPR levels (see Fig. 4). However, neither pooled LPR medians (*p* = 0.165) nor glutamate medians (*p* = 0.939) differed between groups.

### Pooled results over 72 h

Univariate comparison of 72 h data was not significantly different between the no infarction and infarction groups for median LPR (30.7 vs. 40.2; *p* = 0.165), glucose (1.08 mmol/L vs. 0.78 mmol/L; *p* = 0.148) and glycerol (104.0 mmol/L vs. 68.2 mmol/L); *p* = 0.276) as well as for mean PRx (0.07 ± 0.22 vs. 0.08 ± 0.21; *p* = 0.971) (see Table 2). Although the proportion of poor-grade SAH did not differ between groups, exploratory plotting revealed that good-grade SAH patients not responding to treatment presented with a wider variance in LPR. Therefore, it was opted to correct for clinical hemorrhage severity in consequent multivariate analyses.

**Table 2:** Uni- and multivariate comparison of multimodal neuromonitoring data dichotomized by treatment response

	All	No DCI infarction	DCI infarction	Univariate *p*-value^✝^	OR for DCI infarction	95% CI	Multivariate *p*-value^‡^
	n = 63	n = 39	n = 24				
*MMM—72 h*							
CPP (mmHg)—mean ± SD	89.3 ± 21.7	87.6 ± 21.1	91.0 ± 11.3	0.545			
PRx—mean ± SD	0.07 ± 0.21	0.07 ± 0.22	0.08 ± 0.21	0.971	2.70	1.04 to 4.67	< 0.001
PtiO_2_ (mmHg)—mean ± SD	17.0 ± 13.6	16.5 ± 13.0	17.7 ± 14.8	0.783			
Lactate (mmol/L)—Median (Q1 to Q3)	3.28 (2.57 to 5.16)	3.22 (2.47 to 4.13)	4.57 (2.93 to 6.54)	0.284			
Pyruvate (µmol/L)—Median (Q1 to Q3)	93.1 (78.3 to 140.0)	91.9 (78.4 to 122.0)	111.0 (79.9 to 160.0)	0.529			
LPR—median (Q1 to Q3)	32.4 (26.7 to 43.2)	30.7 (25.9 to 35.3)	40.2 (29.2 to 54.2)	0.165	1.02	1.01 to 1.03	< 0.001
Glucose (mmol/L)—Median (Q1 to Q3)	0.873 (0.520 to 1.56)	1.08 (0.593 to 1.67)	0.778 (0.446 to 0.965)	0.148			
Glutamate (µmol/L)—median (Q1 to Q3)	3.98 (1.87 to 9.92)	3.70 (2.05 to 8.59)	4.77 (1.74 to 10.1)	0.939			
Glycerol (mmol/L)—median (Q1 to Q3)	94.4 (52.4 to 139.0)	104.0 (59.1 to 161.0)	68.2 (41.0 to 118.0)	0.276			
Poor-grade SAH (WFNS 3–5)—no. (%)	46 (73.0)	27 (69.2)	19 (79.2)	0.560	1.85*	1.43 to 2.25*	< 0.001
Time (hrs)	n/a	n/a	n/a	n/a	1.02	0.86 to 1.16	0.763

^✝^Univariate analysis based on individual tests of 72 h data including a single mean or median per patient, as described in the methods

^‡^Multivariate analysis based on generalized linear mixed effects modelling with patient level and time, as a random intercept after testing for multicollinearity between predictors via the assessment of the Variance Inflation Factor with a cut-off of 2.5. All continuous data point per patients from the 72 h preceding DCI confirmation were included into the model

^*^Adjusted odds ratios for poor-grade SAH are in relation to the level Yes/1

CPP, cerebral perfusion pressure; hrs, hours; mmHg, millimeter of mercury; µmol, micromole; mmol, micromole; n/a, not applicable; PtiO_2_, brain tissue oxygen tension; PRx, pressure reactivity index; Q1, first quartile; Q3, third quartile; SAH, aneurysmal subarachnoid hemorrhage, SD, standard deviation; WFNS, World Federation of Neurological Surgeons grading scale

### Predictive modeling of treatment response

Based on exploratory plotting, and GAM modeling, PRx, LPR and glutamate were identified as the most promising predictor variables for treatment response. Iterative model building began with a random intercept at patient- and time-level (AIC = 89.1), followed by the gradual addition of fixed-effect predictors. Following the described methodology above, the final model demonstrating the best performance included PRx, LPR, clinical grading and time as fixed effects. The model achieved a pseudo-R^2^ of 27.7% and an AIC of 45.3. Significant main effects were observed for PRx (OR 2.70, 95% CI 1.04–4.67; *p* < 0.001), LPR (OR 1.02, 95% CI 1.01–1.03; *p* < 0.001), and poor-grade SAH (OR 1.85, 95% CI 1.43–2.25; *p* < 0.001). This suggests that, after adjusting for clinical severity, the odds of DCI infarction increase by 10.4% per 0.1 increase in PRx and by 2% per unit increase in LPR. Results are presented in Table 2.

### Effects on functional outcome

A binomial logistic regression model was constructed to identify factors associated with unfavorable outcomes (mRS 0–3). For this purpose, 72-h neuromonitoring results preceding hypoperfusion were pooled. After adjusting for age and WFNS grading, neither PRx (OR 2.16, 95% CI 0.91–5.18, *p* = 0.083) nor LPR (OR 1.01, 95% CI 0.99–1.03, *p* = 0.139) were significantly associated with the dichotomized clinical outcome.

## Discussion

In this prospective cohort study of unconscious patients with subarachnoid hemorrhage, the temporal course of LPR and PRx, although following similar trajectories, was higher in the infarction group, in the hours preceding hypoperfusion. PRx began to increase approximately 24 h before radiological confirmation of hypoperfusion. The LPR demonstrated a dissociation between outcome groups starting 60 h prior to detection of hypoperfusion, with a superimposed more pronounced rise observed in non-responders beginning 24 h prior. Both increased PRx and LPR, after adjusting for clinical hemorrhage severity, were associated with reduced treatment efficacy and the progression to cerebral infarction.

The differences in the time course of pressure-related variables and microdialysis in relation to treatment response could reflect a contrasting spectrum of disease severity. Herein, the interplay between autoregulation and cerebral microdialysis had not been previously investigated. Nonetheless, these results should be interpreted with caution. The effect sizes of both PRx and LPR are small. A ten-point increase in LPR would result in a 20% rise in the odds of DCI-related infarction despite treatment. Taking into account the variance in LPR, decision-making on an individual level might prove difficult. An analysis focusing on the slopes of time trends might prove more useful in individual cases. Additionally, the lack of direct effect on clinical outcome might be due to the relatively small sample of mostly severely affected patients.

The applied treatment strategies are not yet widely accepted and particularly endovascular vasospasm treatment, though yielding promising results [31], remains experimental. Moreover, current hypertensive treatment might exceed the upper limit of autoregulation causing potential harm [39]. In addition, systemic side effects of induced hypertension can occur and these were the main reasons for the premature halt of a randomized trial assessing this treatment for DCI [40]. We do not encourage these results to be interpreted as such that treatment should be withheld from patients with unfavorable PRx and LPR upon diagnosing hypoperfusion. Instead, the question arises of what timely targeted interventions are still possible in order to treat successfully those who would otherwise become non-responders. As for now, no interventional steps for counteracting imparied CA or addressing mitoconfrial dysfunction have been validated in SAH.

### DCI detection in unconscious patients

DCI remains difficult to diagnose in comatose patients. Due to the lack of a clear definition of DCI in the unconscious, the precision and timeliness of diagnosis are compromised. Current neuromonitoring practices rely on absolute thresholds to define pathology; however, these thresholds are extrapolated from traumatic brain injury. A gold standard diagnostic test to confirm suspected DCI does not exist, but imaging techniques are increasingly taking on this role in both research and clinical practice. Treatment strategies where scanning is performed at fixed time points seem ineffective, as the identification of hypoperfusion often occurs too late [41]. Imaging is therefore better utilized as a confirmatory tool in combination with neuromonitoring, where the latter provides the necessary temporal resolution.

Efforts to correlate neuromonitoring data with imaging results have been undertaken previously. As quantified by Xe-CT, lower cerebral blood flow was associated with higher lactate levels and an increase in LPR in patients with SAH who later developed DCI [42]. In a retrospective analysis of 44 poor-grade SAH patients, Tholance et al. observed that low glucose and high LPR predicted the onset of DCI [43]. These changes occurred approximately 67 h before CT detection of infarction. When probes were located within infarcted areas, a surge in glutamate levels was also observed. In contrast, Patet et al. reported that cerebral metabolic abnormalities, such as elevated LPR and reduced glucose levels, became apparent up to 18 h prior to the radiological confirmation of delayed cerebral hypoperfusion [23]. In a positron emission tomography CT study measuring regional cerebral blood flow (rCBF), microdialysis findings correlated with imaging results [44]. While glutamate correlated most strongly with low rCBF, LPR demonstrated high sensitivity and specificity for prolonged ischemic events.

### Interplay between autoregulation and metabolism

Contemporary research has sought to explain DCI through Harper and Glass's dual insult theory [45, 46]. From this perspective, early brain in injury and its consequences—such as hypercoagulation and microthrombi formation, cortical spreading depolarization, blood–brain barrier disruption, and loss of autoregulation—predispose brain tissue to damage. Subsequently, angiographic vasospasm may act as the tipping point, leading to ischemia and infarction. However, given that treatment strategies targeting vasospasm have shown no effect on outcomes [47], and considering the absence of macrovasospasm in a subset of patients who develop DCI-related infarctions [48], a more complex pathological scenario seems likely. This study underlines the role that reduced cerebrovascular reactivity and metabolic dysfunction might play in the pathophysiology of DCI-related infarction. Alternatively, spreading depolarization—detected via either a cortical grid or microdialysis indicating energy depletion—is also brought forward as the underlying pathological mechanism of DCI in patients with impaired autoregulation [49].

### Limitations

Given that CTP imaging was triggered by neuromonitoring results, a self-fulfilling prophecy arises, as measurements exceeding cut-off thresholds often occur prior to imaging. However, this limitation should not impact the comparison between treatment response groups. It is possible that the documented perfusion deficit was present well before its detection—potentially hours earlier. In real-world ICU management, even after the decision for imaging is made, delays due to logistical and organizational challenges in transporting critically ill patients to neuroradiology are common, adding further delays.

This raises the possibility that non-responders may have undergone delayed CTP imaging compared to responders, reaching an autoregulatory or metabolic "point of no return". Conversely, treatment responders may represent cases where imaging was performed promptly, enabling timely interventions. Nonetheless, the observed time window of neuromonitoring changes in this and other studies—ranging between 18 and 72 h—is wide enough to account for these imprecisions while retaining clinical relevance. This timeframe provides an opportunity to take counteractive measures. Therefore, it is likely that hypoperfusion was present before imaging, possibly several hours earlier, but we believe not as far back as 72 h prior to the actual CTP scan.

Apart from PRx, measurements are limited to providing information from within a small region of interest. In an interesting simulation, the likelihood of sensor placement within the vascular territory affected by eventual angiographic vasospasm was highest in lateralized aneurysms and reached a probability of approximately 50% for midline aneurysms [50].

Timely synchronization of physiological data and µD measurements presents a challenge. Due to the perfusion speed of MD catheters, measured values reflect the brain's state in the past. Compensating for this mismatch was deemed unnecessary given that the 17-min transit time from the MD catheter membrane to the collection vial was minor in comparison to the hourly averaging of all physiological data, and this imprecision was considered acceptable.

Finally, as high glycerol levels are generally considered a late indicator of ischemia, reflecting cellular membrane degradation, limited attention was paid to the higher glycerol levels observed in treatment responders during temporal trend analysis [19]. This finding might arise by the outings of a lagging lesion effect of probe placement.

## Conclusion

Loss of cerebral autoregulation and metabolic disturbances seem to precede cerebral hypoperfusion in SAH by up to 24 h or longer. Lower PRx and LPR levels were independently associated with reduced risk of cerebral infarction after treatment. These findings could help identify patients most likely to benefit from treatment, which can be associated with side effects. Further research into more patient-tailored treatment approaches may enhance outcomes in settings characterized by unfavorable metabolic and autoregulatory conditions. 

## Supplementary Information

Additional file1

## Data Availability

The raw data of this analysis can be made available by the authors to any qualified researcher upon reasonable request.. No datasets were generated or analysed during the current study.

## Abbreviations

AIC: Akaike information criterion
ASPECTS: Alberta stroke program early CT score
CI: Confidence interval
CT: Computed tomography
CTP: Computed tomography perfusion scanning
CPPopt: Optimal cerebral perfusion pressure
DCI: Delayed cerebral ischemia
EVD: External ventricular drain
GAMM: Generalized additive mixed model
GCS: Glasgow coma scale
GLMM: Generalized linear mixed-effects model
h/hrs: Hour/hours
ICP: Intracranial pressure
ICU: Intensive care unit
IQR: Interquartile range
L: Liter
Loess: Locally estimated scatterplot smoothing
LPR: Lactate-to-pyruvate ratio
LRT: Likelihood ratio test
mFisher: Modified fisher grading scale
µD: Microdialysis
ml: Milliliter
mmol: Millimole
mmHg: Millimeters of mercury
mRS: Modified rankin scale
OR: Odds ratio
PET: Positron emission tomography
PtiO_2_: Partial pressure of brain tissue oxygen expressed in millimeters of mercury
PRx: Pressure reactivity index
Q1: First quartile
Q1: Third quartile
rCBF: Regional cerebral blood flow
s: Seconds
SAH: Aneurysmal subarachnoid hemorrhage
TCD: Transcranial Doppler sonography
μmol: Micromole
WFNS: World federation of neurological surgeons grading scale
