# Supplementary material for: The metabolic and autoregulatory profile of reversible delayed cerebral ischemia in unconscious patients after aneurysmal subarachnoid hemorrhage: a prospective multimodal neuromonitoring cohort study
Source: Crit Care. 2025 Jun 5;29:228. doi: 10.1186/s13054-025-05460-1 (PMC12142913; doi:10.1186/s13054-025-05460-1)
Supplement: Supplementary file 1 — Additional file1 [file 13054_2025_5460_MOESM1_ESM.docx]

**Supplemental materials**

**Supplemental Figure 1**

**
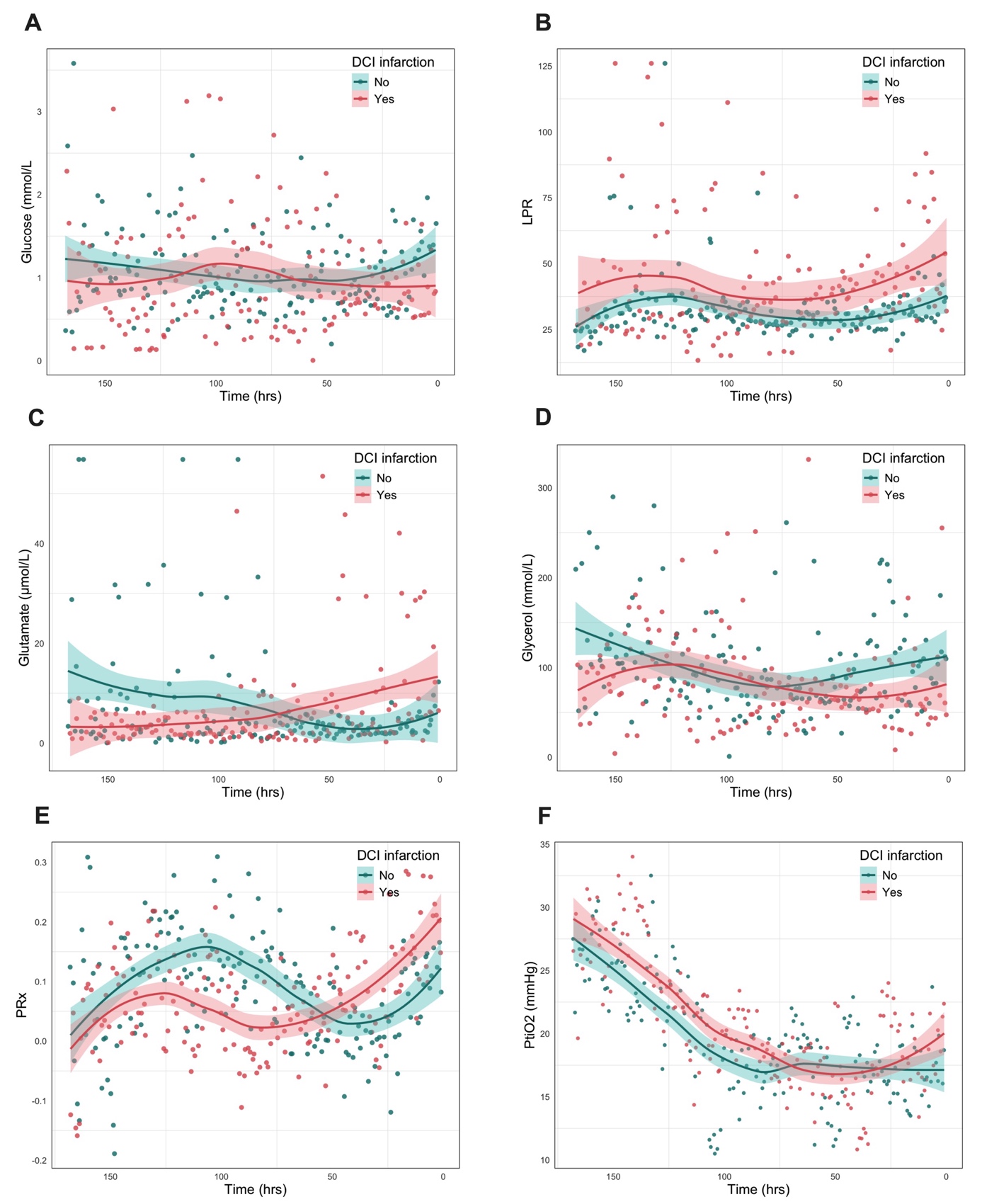
**

**Supplemental Figure 1.** Scatterplot of **A.** median glucose (mmol/L), **B.** median lactate-to-pyruvate ratio (LPR), **C.** median glutamate (µmol/L), **D.** median glycerol (mmol/L), **E.** mean pressure reactivity index (PRx), **F.** median brain tissue oxygen pressure (PtiO_2_ (mmHg)) over 7 days (168 hours) preceding cerebral hypoperfusion and treatment initiation. Data has been dichotomized based on treatment response (development of DCI-related infarction or not). Locally estimated scatterplot smoothing lines are plotted for each outcome group with shaded areas representing 95% confidence intervals.

DCI, delayed cerebral ischemia; hrs, hours; L, litter; mmHg, millimeters of mercury, µmol, micromole; mmol, millimole.

**Supplemental Figure 2**


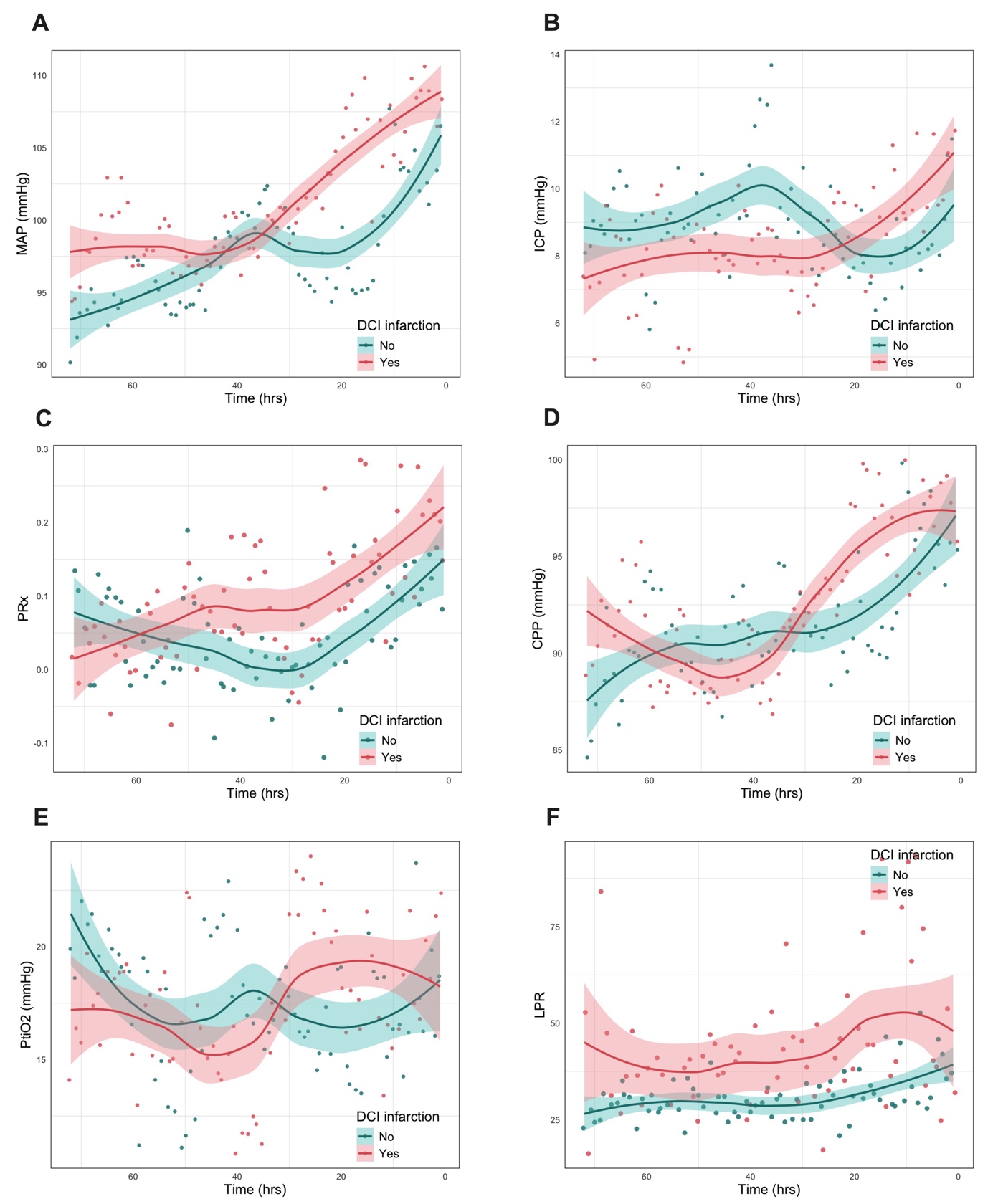


**Supplemental Figure 2.** Scatterplot of **A.** average mean arterial pressure (MAP (mmHg)), **B.** mean intracranial pressure (ICP (mmHg)), **C.** mean cerebral perfusion pressure (CPP (mmHg)), **D.** mean pressure reactivity index (PRx), **E.** median brain tissue oxygen pressure (PtiO_2_ (mmHg)), **F.** median lactate-to-pyruvate ratio (LPR), over 3 days (72 hrs.) preceding cerebral hypoperfusion. Data has been dichotomized based on treatment response (development of DCI-related infarction or not). Locally estimated scatterplot smoothing lines are plotted for each outcome group with shaded areas representing 95% confidence intervals.

DCI, delayed cerebral ischemia; hrs, hours; mmHg, millimeter of mercury.

**Supplemental Table 1**

| **Year** | **Total number of SAH** | **Patients with PtiO2** | **Patients with CMD** | **Patients with INM** | **Proportion of patients with INM (%)** | **Proportion of patients with dual INM (%)** |
| --- | --- | --- | --- | --- | --- | --- |
| 2014 | 31 | 12 | 3 | 12 | 38,7 | 9,7 |
| 2015 | 41 | 24 | 23 | 24 | 58,5 | 56,1 |
| 2016 | 37 | 13 | 13 | 13 | 35,1 | 35,1 |
| 2017 | 31 | 13 | 13 | 13 | 41,9 | 41,9 |
| 2018 | 40 | 21 | 15 | 21 | 52,5 | 27,5 |
| 2019 | 42 | 26 | 22 | 26 | 61,9 | 52,4 |
| 2020 | 46 | 17 | 8 | 17 | 37,0 | 17,4 |
| **Total** | 268 | 126 | 97 | 126 |  |  |

**Supplemental Table 1.** Overview of absolute and relative (%) numbers of patients with invasive neuromonitoring stratified by year of presentation over the study’s inclusion time period from 2014 until 2020.

CMD, cerebral microdialysis; INM, invasive neuromonitoring; PtiO2, brain tissue oxygen saturation measurement; SAH, aneurysmal subarachnoid hemorrhage.
